# Exposure to Dengue Virus During Pregnancy: Incidence and Impact on Maternal and Child Outcomes

**DOI:** 10.4269/ajtmh.24-0387

**Published:** 2024-12-03

**Authors:** Annabelle Smith, Bethel Alebel Bayrau, Caroline Ichura, Jonathan Altamirano, Charles King, Indu Malhotra, Peter Mungai, Francis Mutuku, Dunstan Mukoko, A. Désirée LaBeaud

**Affiliations:** ^1^ Stanford Medicine, Stanford University, Stanford, California;; ^2^ Center for Global Health and Diseases, Case Western Reserve University, Cleveland, Ohio;; ^3^ Department of Environment and Health Sciences, Technical University of Mombasa, Mombasa, Kenya

## Abstract

Dengue virus (DENV) is the most common arbovirus globally, with its incidence growing dramatically in recent decades. Although the effects of DENV infection during pregnancy are unclear, reported associations with adverse health outcomes include miscarriage, prematurity, and low birth weight. In this study, we used an IgG ELISA to identify mothers exposed to DENV during pregnancy by testing samples obtained from a previous study that followed a cohort of pregnant women in Kenya to investigate parasitic infections during pregnancy. We compared adverse pregnancy and infant health outcomes between seronegative mothers and those who seroconverted. Of the 289 participants tested for DENV exposure during pregnancy, we estimated that ∼12 women (4%) would have been exposed to DENV during their gestation period. However, we found that 34 mothers (11.8%) had been exposed to DENV during pregnancy. None of these mothers were hospitalized during pregnancy because of severe DENV infection, suggesting that many may have undergone asymptomatic seroconversion. The demographic risk factors of socioeconomic status, education level, bed net use, and maternal age were not associated with mild or asymptomatic DENV in pregnancy. Although mild or asymptomatic DENV during pregnancy was not associated with late prematurity, reduced postnatal childhood developmental measures, or adverse maternal pregnancy outcomes, we observed an increased risk of low birth weight. The larger-than-expected burden of DENV in pregnancy in this coastal Kenyan cohort and the observed potential risk of low birth weight provide evidence that a more comprehensive study is warranted to fully understand DENV infection during pregnancy.

## INTRODUCTION

Approximately 4 billion people, nearly half of the world’s population, live in areas at risk for dengue, with an estimated 100–400 million infections occurring yearly, although there are discrepancies among these estimates.^1^^,^^2^ In 2023, the highest number of global dengue cases was recorded, affecting over 80 countries in all WHO regions.^3^ Consequently, dengue has become an increasing concern to global travelers.^1^ Dengue virus (DENV) is transmitted to humans via the bite of an infected *Aedes* species, specifically *Aedes aegypti* or* Aedes albopictus* mosquitoes. The incidence of DENV transmission is rising as climate change expands the geographical range of suitable habitats for these mosquitoes, thereby increasing the risk in regions beyond the traditional tropical zones.^1^^,^^3^^–^^5^ DENV transmission peaks at 29°C, whereas for other mosquito-borne pathogens such as malaria, transmission peaks at 25°C.^5^

As with other arboviruses, such as chikungunya (CHIKV) and Zika (ZIKV), DENV infection during pregnancy has reported associations with adverse maternal and pregnancy outcomes. Recent findings indicate that although DENV and CHIKV are widespread, their impact on pregnancy is generally less severe than ZIKV and primarily requires routine prevention measures, whereas ZIKV necessitates more stringent precautions.^6^ The adverse outcomes observed in mothers infected with DENV during pregnancy include maternal mortality, preeclampsia, thrombocytopenia, postpartum hemorrhage, and preterm labor.^7^^–^^10^ Adverse neonatal outcomes, such as infant mortality and low birth weight, have also been reported.^11^^,^^12^ These previous cohort studies have primarily enrolled mothers hospitalized with febrile illness and case studies enrolled mothers hospitalized with DENV.^7^^,^^10^^,^^11^

The incidence of DENV in pregnancy has been estimated in various parts of the world, with estimates ranging from 4% to 10%.^10^^,^^13^^,^^14^ In Sub-Saharan Africa, studies estimating the burden of DENV infection in pregnancy are scarce, despite ongoing outbreaks in the region.^1^^,^^3^^–^^5^ As a result, there is limited epidemiologic evidence characterizing the incidence, transmission dynamics, and clinical outcome of DENV infection during pregnancy and its impact on maternal and neonatal health. This can be attributed to inadequate healthcare infrastructure, limited access to diagnostic tools, underreporting of cases, and a lack of disease surveillance programs and resources for comprehensive research in many areas within the region.^12^^,^^15^ The few studies that have been conducted in settings similar to ours have reported there is no difference in the likelihood of adverse maternal and infant health outcomes among women with and without DENV infection during pregnancy.^16^^,^^17^ These conflicting results necessitate further comprehensive research to accurately determine the impact of DENV infection on pregnancy outcomes.

The purpose of this study was to identify the incidence of DENV among pregnant women in Kenya, where a lack of epidemiological knowledge of DENV has been identified, and determine whether exposure to DENV during pregnancy is associated with various adverse pregnancy or birth outcomes. We studied the demographic characteristics in our maternal cohort to determine if we could identify any risk factors that increase the likelihood of DENV exposure during pregnancy. In addition, we aimed to characterize the differences in maternal and neonatal outcomes between mothers exposed to DENV during pregnancy and mothers who remained unexposed. In characterizing the impacts of exposure to DENV during pregnancy on maternal and child health outcomes, we hope to contribute to the knowledge gap in epidemiologic risk factors to disease and the clinical maternal and neonatal outcomes for DENV in pregnancy.

## MATERIALS AND METHODS

### Subject enrollment.

**Figure 1.**
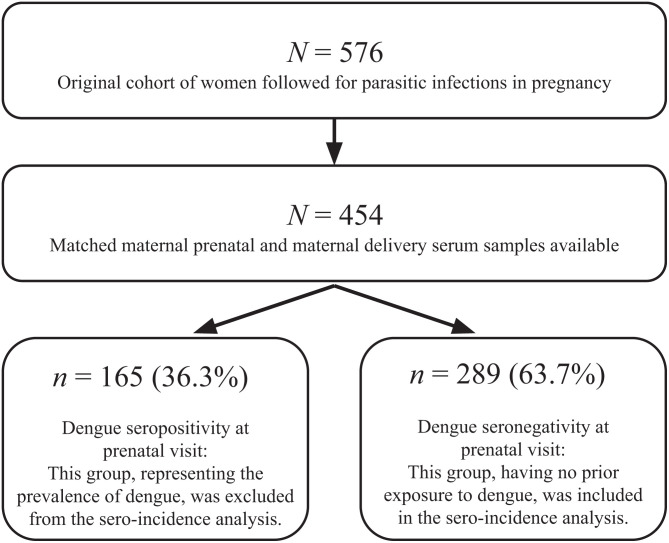
Subject enrollment flowchart. The study was conducted by testing serum samples from an initial cohort of 576 women followed for parasitic infections in pregnancy. After matching maternal prenatal and maternal delivery serum samples, 454 women were enrolled in this dengue in pregnancy study. Statistical analyses of sero-incidence proceeded with the 289 women who were seronegative at their prenatal visit, meaning they did not contract dengue before their first prenatal visit.

Our study used samples obtained from a past prospective cohort study^18^ that followed a cohort of 576 pregnant women for parasitic infection in coastal Kenya from 2013 to 2015 (Figure 1). A total of 122 women were excluded because matched serum samples during the prenatal and delivery phases were not available, and 165 women were excluded from sero-incidence analysis for DENV seropositivity of their prenatal serum sample (Figure 1). Pregnant women were enrolled for the original study at the Msambweni County Referral Hospital antenatal clinic in Msambweni, Kenya, a predominantly rural area on the southern coast of Kenya, and tested for malaria, soil-transmitted helminths, filaria, and *Schistosoma haematobium* during pregnancy. The exclusion criteria of the original study included preterm delivery at less than 34 weeks’ gestation, failure to deliver in the hospital, administration of immunoglobulins or any blood products within the 3 months preceding enrollment in the study, and current active participation in a separate interventional treatment study. Further details of the enrollment of eligible participants, sample collection, and collection of demographic, socioeconomic, pregnancy, and birth information from enrolled mothers have been previously published.^18^ Inclusion criteria for this current sero-incidence study included DENV seronegativity at the first prenatal visit and the availability of matched serum samples during the prenatal and delivery phases (Figure 1) for testing using ELISA to determine exposure to DENV.

### Serological analysis.

Serum samples obtained during the prenatal period and delivery underwent serological testing with an in-house indirect ELISA for the presence of anti-DENV IgG antibodies, as described previously (see the Supplemental Information for detailed ELISA methods).^19^^,^^20^ Seroconversion was defined as having negative serum anti-DENV IgG at the initial prenatal visit and a positive anti-DENV serum IgG at delivery.

## STATISTICAL ANALYSES

To determine the risk factors associated with DENV exposure in pregnancy, we characterized various demographic and lifestyle risk factors in bivariate analyses comparing the associations between mothers without DENV exposure (seronegative) or with DENV exposure (seroconverted) during pregnancy. Fisher’s exact test was used to test this association, with a *P*-value of <0.05 considered significant. The Wilcoxon Mann–Whitney *U* test was used to compare the mean maternal ages between the two maternal groups.

To examine how exposure to DENV during pregnancy affected birth outcomes and neonatal outcomes, bivariate analyses were used to compare maternal birth outcomes and the child health outcomes for mothers without DENV exposure during pregnancy (seronegative) to mothers with DENV exposure during pregnancy (seroconverted). Fisher’s exact test was used to test the association between these health outcomes. Pregnancy outcomes of birth type, negative birth outcomes, preterm birth, maternal anemia, and parasitic infection were assessed. The health outcomes encompassed by the negative birth outcomes variable are fetal demise, meconium aspiration, maternal death, intrapartum fever, severe congenital abnormalities, conditions requiring neonatal resuscitation, and conditions requiring maternal resuscitation. The infant health outcomes studied were low birth weight, length at birth, head circumference, Apgar score, and late prematurity. Late prematurity was calculated as a Ballard score of less than 33, corresponding to less than 37 weeks of gestation.

Child growth outcomes of height, weight, and body mass index were transformed into sex-specific Z-scores by using WHO Anthro software (WHO, Geneva, Switzerland), as previously described.^21^ Z-scores were calculated for child ages at birth, 0–5 months, 6–11 months, and 12–23 months. One-tailed Wilcoxon Mann–Whitney *U* tests were used to analyze differences between Z-scores for children born to seroconverted mothers and children born to seronegative mothers. R Studio software (RStudio Team [2020] RStudio: Integrated Development for R, Boston, MA) ​​was used to perform these Wilcoxon Mann–Whitney *U* tests.

Univariate and ordinal logistic regression models were then used to estimate the odds of health outcomes in pregnant mothers exposed to DENV. Variables that were significant at the *P* ≤0.05 level in the univariate analyses were studied in a multivariate logistic model, with the simultaneous entry of confounders found to be empirically linked to outcomes of interest a priori through literature review. The confounding variables entered in the model were maternal age, anemia in pregnancy, and any parasitic infection during pregnancy. SAS 9.4 (SAS Institute Inc., Cary, NC) was used to perform these statistical analyses.

## RESULTS

**Figure 2.**
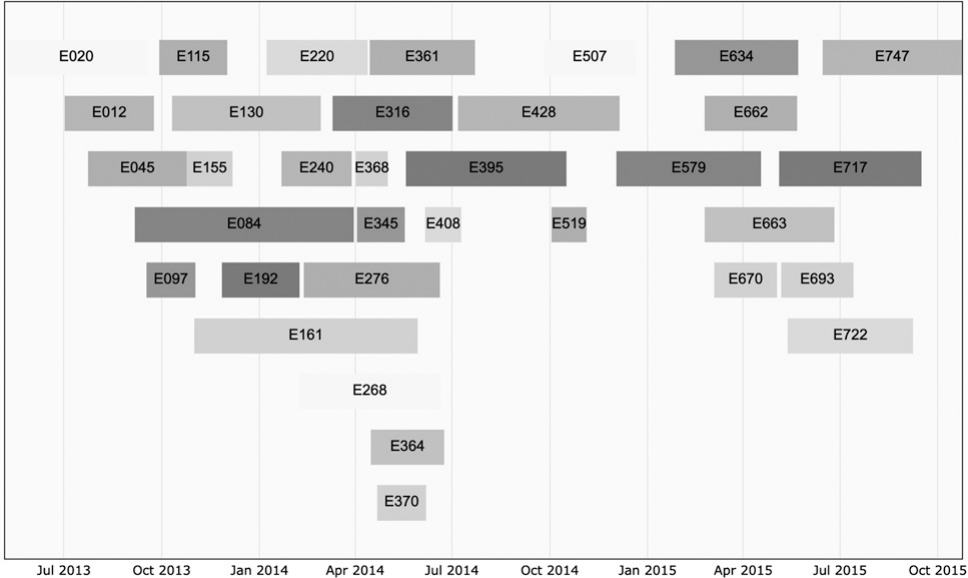
Prenatal visit to delivery date timeline of seroconverters. Each bar represents a seroconverted mother, with the timeline representing the date of prenatal serum sample collection to the final delivery date.

Among the 454 total women in our cohort, the prevalence of dengue at the first prenatal visit was 36.3% (165 women; Figure 1). Of the 289 pregnant women included in this study’s sero-incidence analysis, serological testing with ELISA revealed that 34 women (11.8%) seroconverted during their pregnancy and 255 women (88.2%) remained seronegative at delivery. Pregnancies in which mothers seroconverted were clustered around April 2014 and April 2015, representing probable spikes in DENV transmission (Figure 2). Five out of the 34 DENV-seroconverting mothers were symptomatic, with the following symptoms at their clinic sick visit: fever, cough, chills, running nose, joint pain, stomachache, lower abdominal pain, backache, and headache. At the time of their clinic sick visit, these mothers had temperatures ranging from 35.3°C to 37.0°C.

A lower proportion of pregnant mothers exposed to DENV during pregnancy were found to have low socioeconomic status (47.1% versus 57.3%) and had not attained any level of education (5.9% versus 15.4%), compared with pregnant mothers not exposed to DENV (Table 1). Bivariate analyses demonstrated that maternal exposure to DENV during pregnancy was not significantly associated with demographic factors of socioeconomic status, level of education, or maternal age (Table 1). There was also no significant association between bed net usage and exposure to DENV during pregnancy in our cohort.

**Table 1:** Bivariate analysis of differences in demographic factors between dengue virus-seronegative and dengue virus-seroconverted mothers

Maternal Risk Factor	Number of Seronegative Subjects (%)	Number of Seroconverted Subjects (%)	*P*-Value
Socioeconomic status			
High (≥5,000 Ksh*/month)	109 (42.8%)	18 (52.9%)	0.28
Low (<5,000 Ksh/month)	146 (57.3%)	16 (47.1%)	
Bed net use			
Yes	242 (94.9%)	31 (91.2%)	0.42
No	13 (5.1%)	3 (8.8%)	
Level of education			
None	39 (15.4%)	2 (5.9%)	0.25
Lower primary school	26 (10.2%)	7 (20.6%)	
Upper primary school	143 (56.3%)	17 (50.0%)	
Secondary school	40 (15.8%)	7 (20.6%)	
Unknown	6 (2.3%)	1 (2.9%)	
Maternal age (quartile)			
1: 20 years and younger	81 (31.8%)	8 (23.5%)	0.57
2: 21–24 years old	61 (23.9%)	7 (10.6%)	
3: 25–29 years old	52 (20.4%)	10 (29.4%)	
4: 30 years and older	61 (23.9%)	9 (26.5%)	
Maternal age: mean age (95% CI)	24.47 (24.1–28.8)	24.8 (24.0–25.6)	0.18

* Kenyan shillings.

No significant difference was observed in adverse pregnancy outcomes between women with DENV exposure during pregnancy (baseline seronegative, turned seropositive at delivery) and women with no history of DENV (baseline seronegative and seronegative at delivery; Table 2). No negative birth outcome, encompassing fetal demise, meconium aspiration, maternal death, intrapartum fever, severe congenital abnormalities, conditions requiring neonatal resuscitation, or conditions requiring maternal resuscitation were reported by either group. A higher percentage of malaria infection (8.8% versus 3.5%) during prenatal care was observed among mothers infected with DENV in pregnancy, although this result was not significant.

**Table 2:** Bivariate analysis of pregnancy outcomes between dengue virus-seronegative and dengue virus-seroconverted mothers

Pregnancy Outcome	Number of Seronegative Subjects (%)	Number of Seroconverted Subjects (%)	*P*-Value
Birth type			
Vaginal	241 (96.0%)	32 (94.1%)	0.64
Cesarean	10 (4.0%)	2 (5.9%)	
Negative birth outcomes			
None noted	249 (99.2%)	34 (100%)	0.999
Any negative outcome	2 (0.8%)	0	0.999
Stillbirth	1 (0.4%)	0	0.999
Respiratory distress	1 (0.4%)	0	0.999
Preterm birth (<37 weeks by age)	48 (20.9%)	7 (21.2%)	0.999
Maternal anemia during prenatal care	212 (83.1%)	28 (82.3%)	0.999
Maternal anemia at delivery	183 (73.8%)	27 (79.4%)	0.54
Hookworm during prenatal care	22 (10.4%)	4 (12.5%)	0.76
Roundworm during prenatal care	2 (0.9%)	0	0.999
Malaria during prenatal care	9 (3.5%)	3 (8.8%)	0.15

**Figure 3.**
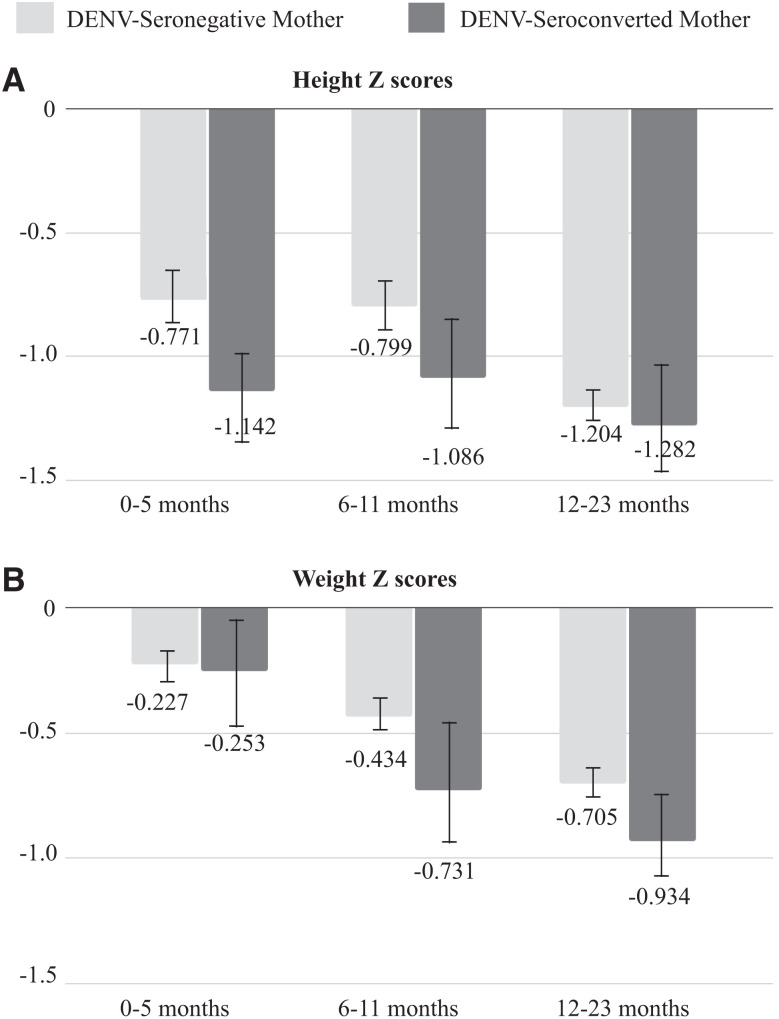
Comparison of anthropometric data between children of dengue virus (DENV)-infected mothers and DENV-uninfected mothers. (**A**) Z-scores of child’s height, as calculated using the WHO Anthro Survey Analyser at 0–5 months, 6–11 months, and 12–23 months. Z-scores were compared via one-tailed Wilcoxon Mann–Whitney tests. Error bars demonstrate the standard error of the mean. (**B**) Z-scores of child’s weight.

Bivariate analyses depict a higher proportion of low birth weight (<2,500 g) among mothers exposed to DENV during pregnancy (Table 3), although this was not statistically significant (*P* = 0.07). Maternal exposure to DENV during pregnancy was not significantly associated with differences in anthropometric factors of length at birth and head circumference or developmental Apgar score between children of DENV-infected and DENV-uninfected mothers (Table 3). Despite children of DENV-exposed mothers having lower height Z-scores on average at 0–5 months, 6–11 months, and 12–23 months (Figure 3), this was not statistically significant (*P* = 0.07, W = 13,684; *P* = 0.10, W = 3,787.5; *P* = 0.09, W = 8,990, respectively). Although not statistically significant, children of DENV-exposed mothers also had lower weight Z-scores on average compared with children of DENV-unexposed mothers (Figure 3) at 0–5 months, 6–11 months, and 12–23 months (*P* = 0.35, W = 12,586; *P* = 0.14, W = 3,714; ​​*P* = 0.15, W = 8,779, respectively).

**Table 3:** Bivariate analysis of birth outcomes between infants of seronegative and seroconverted mothers

Birth Outcome	Number of Seronegative Subjects (%)	Number of Seroconverted Subjects (%)	*P*-Value
Low birth weight (<2,500 g)	23 (9.0%)	7 (20.6%)	0.07*
Length at birth (>2 SD below mean length at birth; <46.1 cm)	17 (6.7%)	4 (11.8%)	0.29
Head circumference (>2 SD below mean head circumference; <31.5 cm)	11 (4.3%)	3 (8.8%)	0.22
Apgar score: 1 minute (score <7)	3 (1.2%)	0	0.999
Apgar score: 5 minutes (score <7)	0	0	0.999
Apgar score: 10 minutes (score <7)	0	1 (2.9%)	0.12
Prematurity (Ballard score <33)	4 (1.6%)	2 (5.9%)	0.15

* Logistic regression *P*-value is 0.045.

**Figure 4.**
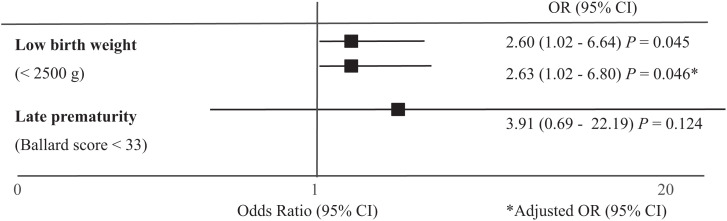
Unadjusted and adjusted odds ratios for adverse neonatal outcomes given dengue virus (DENV) exposure. Odds ratios were calculated with the main exposure as DENV seroconversion during pregnancy. The main outcomes of interest were birth weight (normal weight versus low birth weight of <2,500 g) and late prematurity (gestational age by Ballard score; a score of <33 indicates preterm, corresponding to 37 weeks of gestation). Multivariate analysis of low birth weight adjusted for maternal age, anemia in pregnancy, and any parasitic infection during pregnancy.

In an unadjusted odds ratio analysis, the odds of low birth weight were significantly higher in the maternal DENV exposure during pregnancy group compared with the maternal DENV-unexposed group (crude odds ratio [OR; 95% CI] = 2.60 [1.02–6.64]; *P* = 0.045; Figure 4). In multivariate analysis adjusting for maternal age, anemia in pregnancy, and any parasitic infection during pregnancy, this association between maternal DENV exposure during pregnancy and low birth weight remained significant (adjusted OR [95% CI] = 2.63 [1.02–6.80]; *P* = 0.046; Figure 4). Although prematurity was not significantly associated with DENV exposure in pregnancy in the bivariate Fisher’s exact test, prematurity demonstrated a positive association with DENV exposure in the unadjusted odds ratio analysis (crude OR [95% CI] = 3.91 [0.69–22.19]; *P* = 0.124; Figure 4).

## DISCUSSION

We present a cohort study that characterizes the incidence, health outcomes, and risk factors associated with DENV infection during pregnancy among a Kenyan cohort. Our study helps contextualize the burden of DENV in pregnancy in coastal Kenya, where prospective studies of DENV infection have not been previously conducted to investigate pregnancy outcomes. Using community-based estimates from previous studies in the region,^22^^,^^23^ 12 women (4%) among the 289 pregnant women included in our study were expected to have DENV exposure during pregnancy. However, 34 women (11.8%) in this prospective study were found to have seroconverted for DENV during pregnancy, which is consistent with previous studies finding that DENV incidence is underreported and more widespread than previous data have suggested.^12^^,^^15^​​ Likewise, a high dengue prevalence was found within the study population, with 165 of the 454 total women (36.3%) found to have been exposed to dengue before their first prenatal visit.

There were no officially reported outbreaks during the 2013–2015 study period in Msambweni or the southern Kenyan coast.^24^ However, identified surveillance gaps and our data indicate the possibility that an outbreak may have occurred during the study period. Upon examining the reported symptoms of the seroconverters in this prospective study as they were followed throughout pregnancy, only five of the 34 DENV-seroconverted mothers were found to have reported any symptoms. These symptom reports indicate that the DENV cases in this cohort study were either asymptomatic or exhibited only mild, non-specific symptoms. However, because of the lack of active symptom surveillance, the findings are limited to self-reported symptoms. Nevertheless, high rates of asymptomatic cases may explain the lack of reported outbreaks.

Identifying risk factors for DENV can help address the challenges to treating patients with DENV infection in pregnancy, such as complicated diagnosis due to DENV symptoms being conflated with common pregnancy symptoms of fatigue, nausea and vomiting, and headache.^7^^,^^8^ No significant associations between demographic factors of socioeconomic status, level of education, or maternal age were found with DENV exposure in pregnancy. It is notable that fewer women who experienced DENV exposure during pregnancy tended to be of low socioeconomic status without any level of education, but in our smaller study population, these results were not statistically significant. Elsewhere, previous studies have found that low socioeconomic status is a risk factor for arbovirus infection.^25^^,^^26^ Low income has been identified as a risk factor for pregnant women for CHIKV infection, which is another *Aedes*-transmitted viral fever with similar environmental risk factors.^25^

We aimed to identify differences in adverse pregnancy outcomes, neonatal outcomes, and child developmental outcomes between women with DENV exposure during pregnancy compared with women without DENV exposure during pregnancy. No differences in the mode of delivery or adverse pregnancy outcomes were identified, and there were no cases of maternal death. We excluded women with anti-DENV IgG antibodies at their first prenatal visit to focus on DENV incidence during pregnancy. This excluded secondary DENV infections, which are generally more severe because they are repeat infections. This may explain why our results differ from previous studies linking DENV infection to maternal mortality, preeclampsia, and postpartum hemorrhage.^7^^,^^8^

We identified a higher proportion of low birth weight (<2,500 g) among infants born to mothers exposed to DENV during pregnancy, although this difference was not statistically significant. These infants were 2.60 times more likely to have low birth weight than those born to uninfected mothers. This finding remained statistically significant after adjusting for factors that impact birth weight,^27^^–^^29^ such as maternal age, anemia, and parasitic infections, with a multivariate analysis showing an adjusted odds ratio of 2.63. Because of the initial exclusion criterion of preterm deliveries at less than 34 weeks of gestation, the only preterm births observed in the cohort were between 34 and 37 weeks. Thus, the lower birth weight infants were not born with early prematurity, suggesting a potential association between DENV and growth restriction, although including births before 34 weeks might influence this observation. Although we could not determine if exposure to asymptomatic or mild DENV affected early preterm birth in our study, we observed that infants born to mothers exposed to DENV during pregnancy were 3.91 times more likely to experience late prematurity, although this was not statistically significant. However, because no significant differences in child anthropometric measures or developmental scores were identified, it appears that asymptomatic or mild DENV in pregnancy is not associated with notably adverse underdevelopment of the infant at birth. Consistent with this finding, children of DENV-exposed mothers had lower postnatal (up to 24 months of age) height and weight Z-scores on average, but there were no statistically significant differences.

This study has several limitations. The exclusion criteria of failure to deliver in the hospital and early preterm birth (<34 weeks) limited our study size and ability to form definitive conclusions. Mothers with a pregnancy result of stillbirth (away from the hospital) or early preterm birth would not have been included in our data analysis, although their outcomes may have been relevant. In all, 12.7% of mothers enrolled in the original study were excluded or lost to follow-up because of delivery at a non-study site, delivery samples not taken, early infant death, early preterm birth, antenatal maternal death, or withdrawal from the study.^18^ The small sample size of 289 limited the ability to form comprehensive conclusions about associations with demographic factors and adverse health outcomes. Further limitations include a lack of generalizability because our study was performed only in coastal Kenya. In the future, larger studies with more diverse populations can help to clarify risk factors for DENV exposure during pregnancy.

In our cohort, exposure to DENV during pregnancy represented a substantial burden, with an incidence of nearly 12%. Given the symptom profile of the DENV-exposed women suggests asymptomatic DENV, this study is consistent with published literature that maternal and neonatal health impacts are minimal with asymptomatic DENV.^16^^,^^17^ The potential risk of low birth weight is indicated by the higher odds observed among children born to mothers with DENV during pregnancy. This finding underscores the importance of monitoring pregnant women at risk of DENV infection because low birth weight can have long-term implications, including an increased risk of chronic diseases in adulthood, such as hypertension.^30^ In contrast, DENV does not seem to be linked to more adverse pregnancy or child health outcomes, offering reassurance to pregnant women at risk of DENV infection and their healthcare providers. Travelers to DENV-endemic regions can be informed that more severe health complications appear unlikely with asymptomatic or mild DENV during pregnancy. This finding is also consistent with previous reports that DENV infection in Africa is less symptomatic and severe than in other DENV-endemic areas.^31^^,^^32^ Mild and asymptomatic dengue in Africa is significantly underreported, however, aligning with our finding of higher-than-expected DENV incidence and prevalence in our maternal cohort. High rates of asymptomatic DENV in Africa, contrasting with the more severe DENV observed in the Americas or Asia, highlight challenges with DENV diagnosis and the current clinical criteria in Africa.^33^

DENV is an arbovirus at the forefront of global public health efforts. Although DENV epidemiology is well-studied in Asia and the Americas, the virus’s impact in Africa remains unclear. Our study contextualizes the incidence, impacts, and risk factors of DENV for pregnant women in Kenya. Dengue infections in Kenya seem to be less severe than in other world regions, and this may prevent the disturbing pregnancy and neonatal outcomes seen elsewhere. Because DENV outbreaks will continue in the future as climate change creates an environment more conducive to DENV transmission, understanding the burden, impacts, and risk factors of DENV in pregnancy is imperative to direct public health efforts of vector control, community education, and management of the disease. Public health officials and arbovirus researchers should advocate for future studies analyzing DENV infection during pregnancy to execute public health initiatives that optimally combat DENV.

## Supplemental Materials

10.4269/ajtmh.24-0387 Supplemental Materials
