# Supplementary material for: Exposure to Dengue Virus During Pregnancy: Incidence and Impact on Maternal and Child Outcomes
Source: Am J Trop Med Hyg. 2024 Dec 3;112(2):396–402. doi: 10.4269/ajtmh.24-0387 (PMC11803650; doi:10.4269/ajtmh.24-0387)
Supplement: Supplemental Materials [file tpmd240387.SD1.pdf]

Supplementary Methods

Maternal prenatal and maternal delivery serum samples underwent serological testing by indirect enzyme-linked immunosorbent assay (ELISA) for the presence of anti-DENV IgG antibodies, as described previously (40,41). DENV1-4 antigen derived from four serotypes (DENV1: Western Pacific 74; DENV2: S16803; DENV3: CH53489; and DENV4: TVP360) was used to coat nunc-immuno 96-well plates. Following coating overnight at 4 ºC, plates were washed with a phosphate buffered solution (PBS)/0.01% Tween-20/0.01% NaN3 wash buffer, blocked with blocking buffer (5% powdered milk in PBS) for two hours at 37 °C, and washed before adding diluted samples. Aliquots of 50 µL of serum dilutions (1:100 dilutions in blocking buffer) were added to appropriate wells. Plates were incubated overnight at 4 ºC and subsequently incubated for an hour at 37 ºC.

Next, 50 µL of secondary antibody (1:2000 dilution, goat anti-human IgG conjugated to alkaline phosphatase, Jackson ImmunoResearch 109-055-008) was added and plates were incubated at 37 °C for one hour. Following incubation, plates were washed and 100 µL of alkaline phosphatase substrate (Thermo Fisher Scientific CAS 264-83-9) in PnPP buffer (1 ug/1 ml) was added before incubation at 37 ºC for 30 minutes. After the incubation, the optical density of the plates was read at 405 nm (BioTek Gen5.0 software). Cut-off values for positivity were defined as at least half of the value of the positive control OD and for negativity less than twice the value of the negative control OD, as described in previous protocols.[19,20] Controls utilized in these assays were previously confirmed by confirmatory plaque reduction neutralization tests (PRNT). Seroconversion was defined as having negative serum anti-DENV IgG at the initial prenatal visit and a positive serum anti-DENV IgG at the delivery.
